# Supplementary material for: Integrated Clinical, Molecular and Immunological Characterization of Pulmonary Sarcomatoid Carcinomas Reveals an Immune Escape Mechanism That May Influence Therapeutic Strategies
Source: Int J Mol Sci. 2023 Jun 23;24(13):10558. doi: 10.3390/ijms241310558 (PMC10341842; doi:10.3390/ijms241310558)
Supplement: Supplementary file 1 [file ijms-24-10558-s001.zip › Supplementary Figure S1.pdf]

Supplementary Figure S1. Proportion of PD-L1 Expression in Patients with Targetable Genomic Alterations

PD-L1 TPS		TPS < 1%		TPS 1 - 49 %		TPS ≥ 50%	
	all	n	%	n	%	n	%
EGFR (p.L858R; p.E709K)	2	0	0	2	100	0	0
KRAS G12C	25	3	12	3	12	19	76
MET Ex14skip	24	6	25	6	25	12	50
BRAF V600E	2	0	0	1	50	1	50
CCDC6-RET fusion	3	0	0	2	67	1	33
EML4-Alk (E13A20) fusion	2	1	50	0	0	1	50
