# Supplementary material for: Integrated Clinical, Molecular and Immunological Characterization of Pulmonary Sarcomatoid Carcinomas Reveals an Immune Escape Mechanism That May Influence Therapeutic Strategies
Source: Int J Mol Sci. 2023 Jun 23;24(13):10558. doi: 10.3390/ijms241310558 (PMC10341842; doi:10.3390/ijms241310558)
Supplement: Supplementary file 1 [file ijms-24-10558-s001.zip › Supplementary Figure S2.pdf]

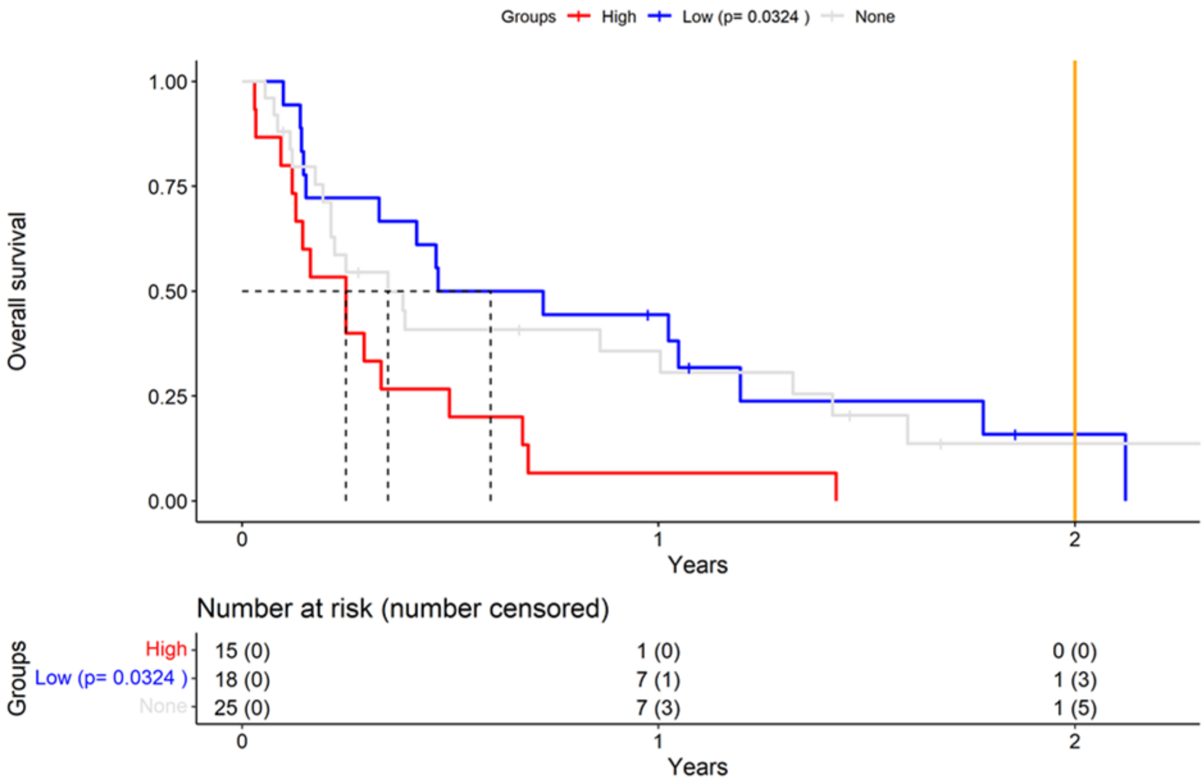

Supplementary Figure S2. Kaplan-Meier plots displaying correlations of processing mutations to OS in the sarcomatoid NSCLC cohort. Only patients with advanced-stage tumour disease (IIIB-IV) were included. A high mutation count constitutes more than one mutation, while low is equivalent to just one mutation. Dotted lines represent the medial OS of each group. The p-value was calculated by score-logrank test.
