# Supplementary material for: Integrated Clinical, Molecular and Immunological Characterization of Pulmonary Sarcomatoid Carcinomas Reveals an Immune Escape Mechanism That May Influence Therapeutic Strategies
Source: Int J Mol Sci. 2023 Jun 23;24(13):10558. doi: 10.3390/ijms241310558 (PMC10341842; doi:10.3390/ijms241310558)

Supplementary Figure S3. Patients who have received ICI show better overall survival when no or few processing mutations are present compared to patients who have many processing mutations ($p < 0.05$). Though, these results should be interpreted with caution, as of the 10 patients treated with ICI, only four patients received monotherapies with pembrolizumab. **3A**. Entire cohort, stage I-IV. **3B**. Advanced stages IIIB-IV only.

S3A.

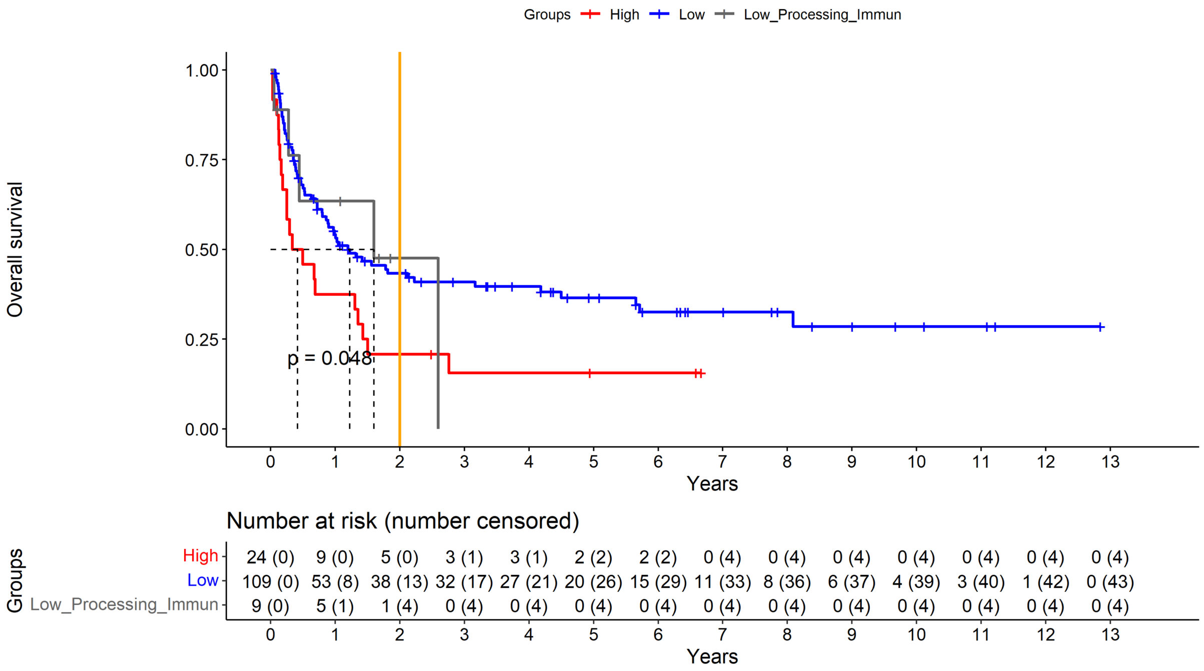

S3B.

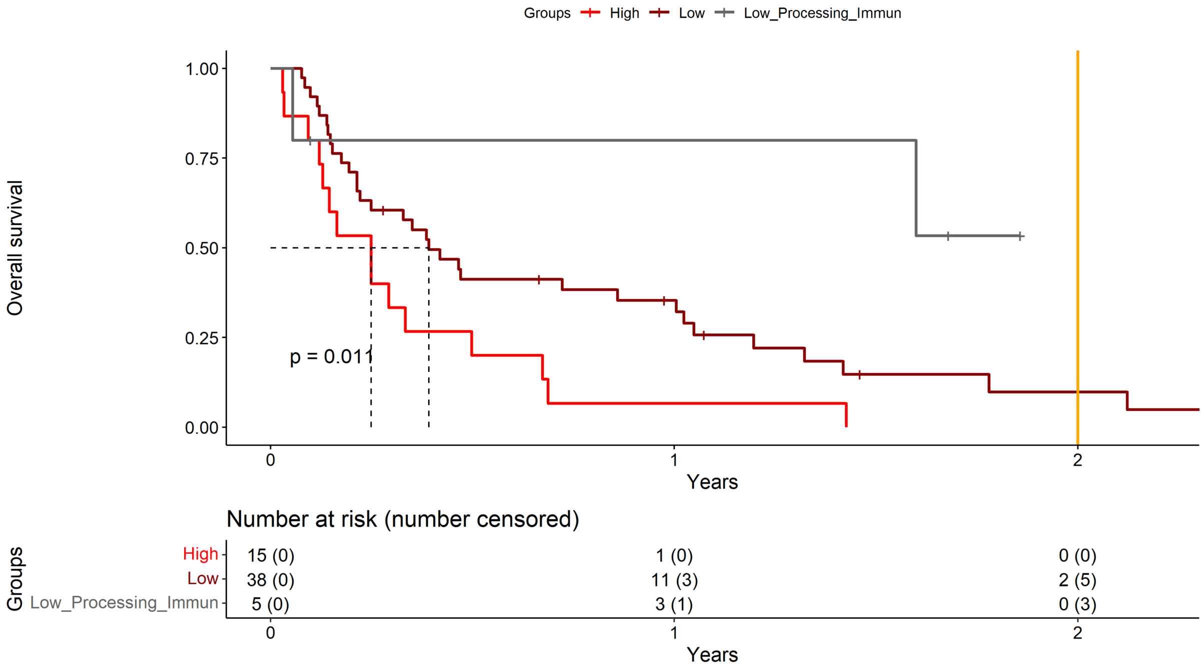

Supplement: Supplementary file 1 [file ijms-24-10558-s001.zip › Supplementary Figure S3.pdf]
